# SRAssembler: Selective Recursive local Assembly of homologous genomic regions

**DOI:** 10.1186/s12859-019-2949-4

**Published:** 2019-07-02

**Authors:** Thomas W. McCarthy, Hsien-chao Chou, Volker P. Brendel

**Affiliations:** 1 Department of Biology, Indiana University, Bloomington, 47405 Indiana USA; 2 Department of Oncology, St Jude Children’s Research Hospital, Memphis, 38105 Tennessee USA; 3 Department of Computer Science, Indiana University, Bloomington, 47405 Indiana USA

**Keywords:** Local assembly, NGS assembly, Genomics, Homolog

## Abstract

**Background:**

The falling cost of next-generation sequencing technology has allowed deep sequencing across related species and of individuals within species. Whole genome assemblies from these data remain high time- and resource-consuming computational tasks, particularly if best solutions are sought using different assembly strategies and parameter sets. However, in many cases, the underlying research questions are not genome-wide but rather target specific genes or sets of genes. We describe a novel assembly tool, SRAssembler, that efficiently assembles only contigs containing potential homologs of a gene or protein query, thus enabling gene-specific genome studies over large numbers of short read samples.

**Results:**

We demonstrate the functionality of SRAssembler with examples largely drawn from plant genomics. The workflow implements a recursive strategy by which relevant reads are successively pulled from the input sets based on overlapping significant matches, resulting in virtual chromosome walking. The typical workflow behavior is illustrated with assembly of simulated reads. Applications to real data show that SRAssembler produces homologous contigs of equivalent quality to whole genome assemblies. Settings can be chosen to not only assemble presumed orthologs but also paralogous gene loci in distinct contigs. A key application is assembly of the same locus in many individuals from population genome data, which provides assessment of structural variation beyond what can be inferred from read mapping to a reference genome alone. SRAssembler can be used on modest computing resources or used in parallel on high performance computing clusters (most easily by invoking a dedicated Singularity image).

**Conclusions:**

SRAssembler offers an efficient tool to complement whole genome assembly software. It can be used to solve gene-specific research questions based on large genomic read samples from multiple sources and would be an expedient choice when whole genome assembly from the reads is either not feasible, too costly, or unnecessary. The program can also aid decision making on the depth of sequencing in an ongoing novel genome sequencing project or with respect to ultimate whole genome assembly strategies.

**Electronic supplementary material:**

The online version of this article (10.1186/s12859-019-2949-4) contains supplementary material, which is available to authorized users.

## Background

Advances in next-generation sequencing (NGS) approaches have dramatically changed access to genome data, not only with respect to reference sequencing for many species, but increasingly for population studies of genomic variation (e.g., [1–3]. Applications of NGS include the creation of detailed maps of genetic variation [4–6], DNA methylation [7, 8], and transcription factor binding sites [9, 10].

Because NGS relies on extensive sequence coverage with small reads, accurate assembly of the reads into large contigs, scaffolds, and pseudochromosomes is an intrinsic part of the approach, and many NGS assembly tools have been developed for this purpose. Based on de Bruijn graphs [11], programs like Velvet [12], ABySS [13], ALLPATH [14], and SOAPdenovo2 [15] have been shown to effectively handle millions of short reads. Currently, research on genome assembly focuses on reducing error rates and increasing contig sizes, usually evaluated by N50 (at least half the assembled nucleotides are part of contigs of length N50 or longer) [16]. Strategies to improve quality include gene-boosted [17] and homology-guided assembly [18], which use existing information from related sequences to improve assembly results.

Despite advances in assembly software, assembling the massive amount of short read data necessary for de novo genome assembly is still a difficult technical task [19]. For eukaryotic genomes, de novo assembly typically requires high-performance computing resources with large memory and fast processors. Even with such extensive resources, it may take hours or days for completion of a single assembly attempt. If the resulting assembly is not satisfactory, parameter adjustments for subsequent runs and comparative evaluation of different draft assemblies are typically required. All of these challenges must ultimately be overcome to get a reliable whole-genome assembly.

However, whole-genome assembly is not necessarily the immediate, nor the only, goal of genome-wide NGS approaches. Because of the cost-effectiveness of NGS technologies, a research group may well choose genome-wide NGS for a species even if they are interested in only a subset of the species’ genes: for example, homologs of genes already identified in other species as being involved in a specific biochemical pathway or cellular structure. Alternatively, researchers can take advantage of the petabases of sequencing reads already present in the International Nucleotide Sequence Database Collaboration Sequence Read Archive (SRA) [20], which may include read deposits of interest for which no publicly available whole genome assemblies are available. Pre-assembly stages of massive read collections will also likely be a component of data release from large-scale sequencing projects such as the Earth BioGenome Project [21]. In these cases, it becomes expedient to restrict the assembly to the genic regions of interest; that is, instead of assembling the entire genome, assembling only the reads which correspond to annotated homologous genes of interest. By limiting the assembly to specific genomic regions, required resources and running time can be drastically reduced, and interpretation of output can be easily focused on the desired comparison of homologous regions. In pursuit of this goal, we have developed the program SRAssembler (Selective Recursive local Assembler).

**Fig. 1:**
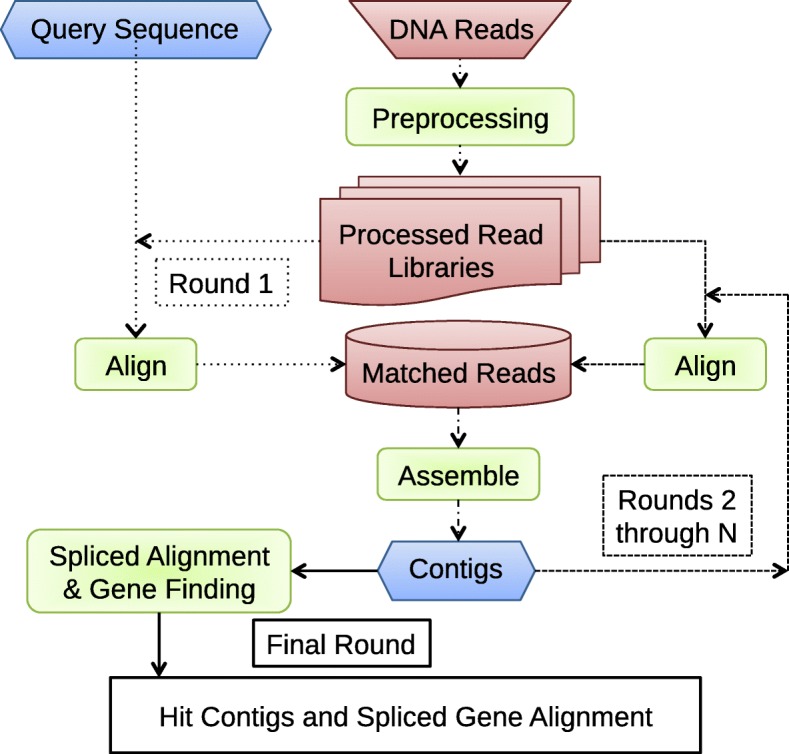
SRAssembler workflow. The pipeline takes as input a query sequence and DNA sequencing read files, which are split into smaller files in a preprocessing step, allowing for parallelization of alignments. In the initial round, SRAssembler aligns the processed reads against the query (DNA or protein) sequence to identify reads that could potentially contribute to a homologous contig. Matched reads are then assembled into contigs, which are used as queries in the next round of searching the read libraries. The reads found in recursive rounds are reassembled, extending the edges of the contigs until no further matching reads are found or until user criteria for success are met (usually a finite number of rounds or complete coverage of the query). In the final round a spliced alignment program aligns the original query to the final contigs, predicting contigs that are good matches for the query and their homologous gene structure(s)

SRAssembler uses a protein or DNA sequence from a related species as a query input to select and assemble NGS reads from a sequencing project in a different species or individual of interest (Fig. 1). Reads that are potentially homologous to the query sequence are assembled into contigs that serve as queries for the next recursive round of searching the reads, representing an “in silico” chromosome walking strategy as originally developed for mining the now outdated NCBI Trace Archive with the Tracembler program [22]. The user specifies success criteria that determine the break condition for the recursion, and at the last stage, the original query is aligned against the assembled contigs using spliced alignment software to identify potential gene structures.

Searching for matching reads based on the sequence of assembled contigs is a strategy also used by assembly gap-filling software such as GapFiller [23]. Indeed, SRAssembler could also be used for specific gap-filling simply by providing the sequences surrounding the gap as a target. What distinguishes SRAssembler is the use of local de novo assembly of matching reads rather than genome-wide reference-based assembly, as well as the ability to use cDNA and protein probes for targeted assembly.

SRAssembler will also be useful to explore parameter spaces for whole genome assembly in a time-efficient manner. For a set of genes strongly expected to be present in a genome currently being sequenced, different assembly parameters can be explored and evaluated as to completeness of assembly of this diagnostic set of genes. The best parameter set can then be used for whole genome assembly. Similarly, completeness of assembly of such a gene set may also give insight into any additional sequencing needed for sufficient coverage of a novel genome of unknown size. If all the diagnostic genes can be locally assembled, then current coverage is likely adequate, whereas incomplete assembly may indicate the need for additional sequencing.

We illustrate the functionality of SRAssembler with examples using both simulated and genuine sequencing reads. We compare the efficacy of SRAssembler relative to whole-genome assembly and demonstrate that SRAssembler can not only assemble the likely orthologous gene, but can also distinguish and recover related paralogous genes using a single query. We show how the tool can be used to study gene body and promoter region variation using population genome data that are available merely as relatively low coverage, unassembled read deposits.

## Implementation

SRAssembler is implemented as a C++ program that relies on a number of freely available external programs for string matching, assembly, and spliced alignment. Default minimal prerequisites are Vmatch [24], SOAPdenovo2 [15], and GenomeThreader [25]. The program can be compiled with any standard C++ compiler, or under openMPI for a multi-processor parallel computing platform[26]. Because SRAssembler is implemented within an object-oriented framework, advances in alignment and assembly software can be easily incorporated as new options within the workflow. Currently SRAssembler supports contig assembly with SOAPdenovo2 or ABySS, spliced alignment with GenomeThreader or GeneSeqer [27], and de novo gene finding with SNAP [28].

### In silico chromosome walking strategy

The basic strategy implemented in SRAssembler is depicted in Fig. 1. Initially, NGS reads are aligned to a query sequence using the fast string matching program Vmatch. Query sequences can be either protein or DNA sequences provided in FASTA format. If the query sequence is a protein, the matching is to all possible translations of the reads (Vmatch option -dnavsprot).

Retrieved reads from the initial matching are assembled into contigs that become query sequences for subsequent rounds of in silico chromosome walking. Thus, in each round of the workflow, larger sets of presumed relevant read are retrieved from the input and assembled until one of alternative stopping criteria are met (see below), at which point the assembled contig(s) will contain the sought homologous gene or the program declares failure of the search within the given criteria. In cases when read coverage is expected to be low or when only short contigs were assembled in round 1 for later searches, SRAssembler can be run with the command-line ‘-a’ flag to set a later round to begin assembling found reads into contigs. Until that round is completed, reads found by SRAssembler will be used directly as queries, allowing reads that could not be assembled into contigs a better chance of finding overlapping reads.

### Preprocessing reads

Input read files can be in either FASTQ or FASTA format and single-end or paired-end. If a read library is paired-end, the reads must be in two sorted files rather than a single interleaved file. Although SRAssembler accepts single-end reads, paired-end reads typically provide better results because they allow reads not matching exons to be found more quickly. SRAssembler supports assembly from multiple read libraries simultaneously.

SRAssembler can take advantage of multiple processors to parallelize the search for new reads using the Message Passing Interface (MPI) protocol. To facilitate this, input reads data are split into several chunks. Each chunk is indexed by Vmatch, allowing very fast searching of the reads for matches to query contigs. These processed reads can be used again for subsequent SRAssembler runs.

### Read assembly

At the end of each workflow round, after searching for new reads with Vmatch, SRAssembler assembles all the reads it has found so far into new contigs. By default, SRAssembler invokes SOAPdenovo2 for the assembly step. The ABySS assembler may be used instead at the user’s discretion. During the assembly step, the assembler is run multiple times with different k-mer values (the default setting uses 15, 25, 35, and 45). The contigs of each assembly are compared to the query sequence using spliced alignment software (by default, GenomeThreader, with the option of GeneSeqer also available). The k-mer size that produces the greatest spliced alignment length is considered to be the best k-mer of that round, and the contigs produced by that k-mer will become the query sequences for the next round. Before the contigs are used, very short contigs (by default shorter than 200 bp) are removed, and low complexity regions of the remaining contigs are masked with NCBI’s DustMasker [29].

### Cleaning non-matching contigs and reads

Periodically (every four rounds, by default), assembled contigs and found reads will be culled of non-matches, which can slow SRAssembler and impact the results. During these “cleaning rounds,” assembled contigs are matched against the original protein or DNA query using Vmatch, and any contigs that do not have at least partial matches to the query are discarded. This can happen, for example, when the queries contain repetitive sequences that would match elsewhere in the genome apart from their occurrence in the gene of interest. After non-matching contigs have been removed, all of the reads that have been found so far are matched to the remaining contigs, and any reads that do not match (and therefore were not assembled into the matching contigs) are also discarded. SRAssembler will also perform cleaning at the end of a round that produces a number of contigs higher than a threshold (default 500 contigs). This is to prevent slowdown caused by the assembly of a highly duplicated region leading to an excess of contigs and reads unrelated to the query.

Cleaning non-matching contigs and reads improves the speed of SRAssembler and can prevent extraneous reads from interfering with the assembly of high quality contigs that match the query. However, the cleaning can also remove useful reads that would have been assembled into a matching contig in a later round, potentially preventing a hit contig from being extended. Adjusting the frequency of cleaning can be useful when attempting to extend a hit contig to more completely cover a region of interest, or when trying to find or complete additional paralogous sequences.

### Contig maximum length

If an assembled contig is larger than the predefined maximum contig size (default 10,000 bp), that contig will be removed as a query for future rounds. The head and tail of these contigs are trimmed to make their size equal to the defined maximum contig size, and then are copied to the candidate-long-contig file. In the next round, any additional matching reads found by Vmatch using the contigs that did not exceed the maximum length are added to the pool of found reads. If long contigs assembled in this round match the candidate long contigs from the previous round (that is, the long contigs from the previous round are assembled again even with the addition of new reads), those candidate long contigs are moved to the permanent long contig file. The pool of matched reads is aligned to the permanent long contig file and any matching reads are removed from the pool in order to speed subsequent assemblies and prevent the long contigs from being extended any further. These long contigs are retained until recursion stopping criteria are met and are included in the final contig file.

### Stopping criteria

The recursion is terminated as soon as one of the following criteria is met:

(1) Success – a hit contig is found. Here, a “hit contig” is defined as an assembled contig that satisfies the current user-set criteria for success: the contig length matches or exceeds the minimum specified value (default 200 bp); the spliced alignment similarity score of query versus contig is greater or equal to the threshold set (default 0.5); and the extent of the spliced alignment covers at least the specified minimum fraction of the query (default 0.8).

Alternatively, the assembly attempts will be stopped short of success in case of:

(2) No new reads can be found, meaning no contigs can be further extended.

(3) A specified maximum number of iterations is reached.

(4) All assembled contigs match or exceed the specified maximum length.

To determine if criterion 1 is met, the spliced alignment program is used to map the original query onto each round’s assembled contigs. Criterion 1 can be ignored with the ‘-f’ flag, forcing SRAssembler to complete the user-specified maximum number of rounds, potentially extending hit contigs beyond the boundaries of the homologous gene. Alternatively, the ‘-E’ option can be used to compel SRAssembler to complete extra rounds after criterion 1 is met. These may be helpful if the user wants to extend assembled contigs into regions flanking the homologous coding region, but in some cases using the ‘-f’ or ‘-E’ options may lead to lower quality hit contigs, as extraneous reads can disrupt assembly in later rounds.

When SRAssembler cannot find a contig that meets the success criteria, it will run until one of criteria 2, 3, or 4 is met. This can occur because the source of the reads does not contain a homolog to the probe, because the success criteria are too stringent, or because of any of the various factors that affect read assembly.

### Final round

After recursion is terminated, the contigs assembled in the final round that are longer than the minimum length are reported in the “all_contigs.fasta” file. Spliced alignment of the contigs assembled in the final round is used to create the “hit_contigs.fasta” that meet the user-specified criteria (mentioned above), and the spliced alignment file is available as “output.aln”. If the probe contains a common protein domain, the “all_contigs.fasta” file may contain contigs that include that domain, but they will not be included in the “hit_contigs.fasta” file unless they meet the user-specifiable criteria for a good match. Optionally, an *ab initio* gene finding program (currently SNAP is supported) will attempt to identify potential gene structure in the hit contigs and produce “output.ano”, in addition to and independent of the spliced alignment.

## Results

SRAssembler can be installed from our github repository https://github.com/BrendelGroup/SRAssembler, which also includes the manual and detailed instructions for installing prerequisite third-party software. Scripts to download the data used and to perform all of the analyses reported in this paper are included as Additional file 3. The simplest way to run SRAssembler on any single or multi-processor Linux system is via its containerized version as a Singularity image [30], which has all prerequisites bundled. The Singularity image of SRAssembler is available at Singularity Hub [31] at https://www.singularity-hub.org/collections/1653. SRAssembler version 1.0.0 was used for the experiments in this manuscript.

### Assembly of homologous loci from simulated data

The goal of the SRAssembler strategy is to construct local assemblies of NGS reads that encode putative homologs of a query protein or cDNA sequence. Because of our own expertise in plant genomics, our SRAssembler illustrations are mostly reported with plant genomic examples. The program is agnostic to the genomic sources of the reads, although parameter settings may have to be adjusted to fit characteristics of the genome.

To demonstrate the SRAssembler strategy, we used the rice protein sequence Os07g26940.1 as a query to try to assemble a contig containing the known homologous gene At1g01230 from simulated sequencing reads from Arabidopsis chromosome 1. We simulated paired-end NGS sequencing using the SAMTools program wgsim [32]. The number of reads N was calculated as N = (length of chromosome 1 x coverage) / (length of reads x 2). Parameters were set as follows: base error rate 0.02, mutation 0, and fraction of indels 0.10. Read length was set to 70 bp, and insert size to 340 bp with standard deviation 50 bp.

**Fig. 2:**
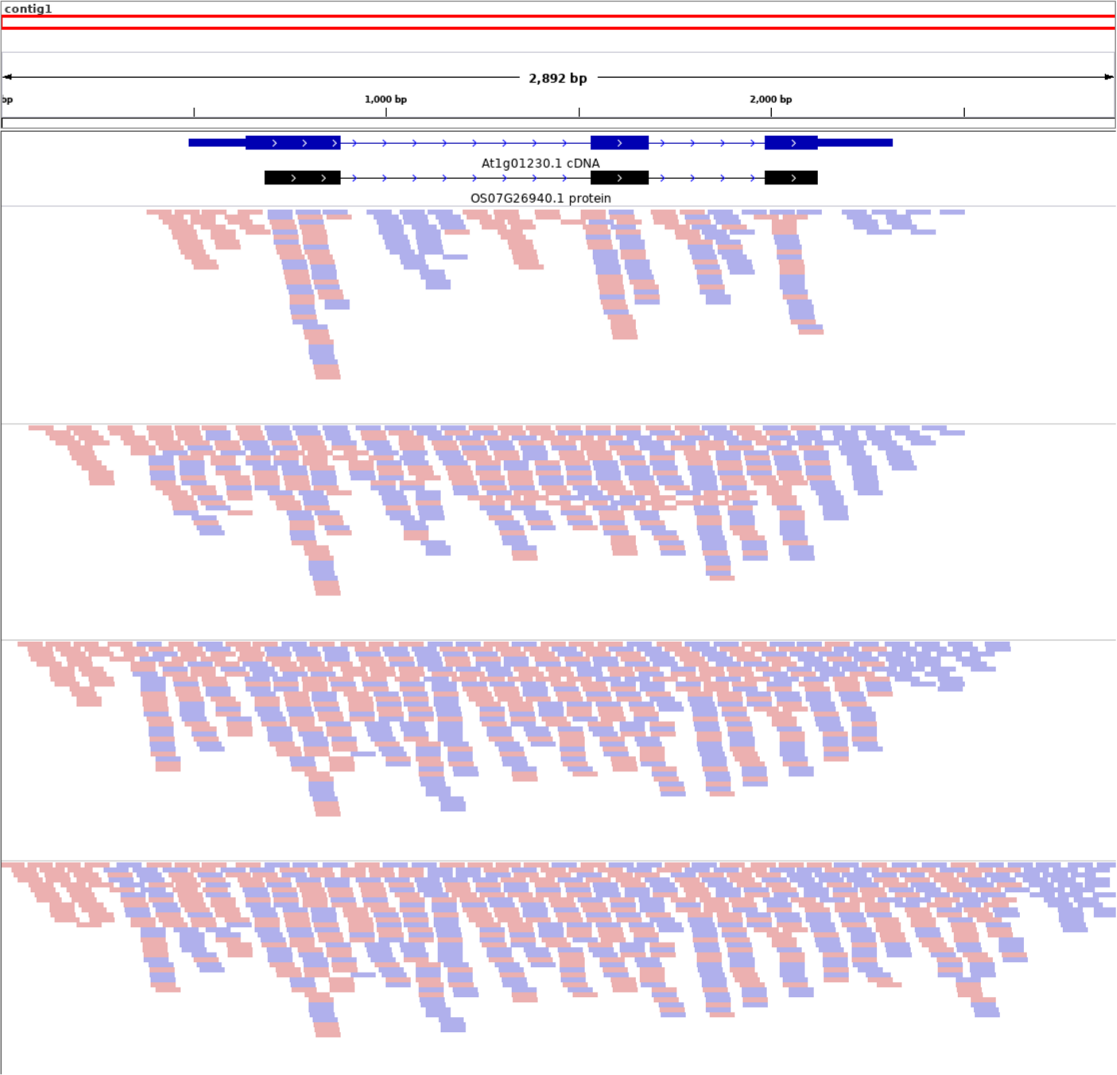
Assembly of At1g01230 using the SRAssembler strategy. The cDNA sequence of At1g01230.1 (blue) and the protein sequence of Os07g26940.1 (black) are shown aligned to the final contig (red) produced by SRAssembler after 4 rounds of assembly using reads data simulating 20X coverage of Arabidopsis chromosome 1. The reads found in each round of the SRAssembler run of At1g01230 are also mapped onto the final contig. Mapped reads are shown as pink or blue rectangles, representing forward and reverse orientation relative to the contig. In the initial round, reads were identified that align with each exon of the query protein. Because we simulated paired-end reads, some of the mapped reads align to the introns of the target gene. The mapped reads become the query contigs for subsequent searches of the read library, “walking” further into the introns and out along the chromosome

Figure 2 depicts the gene structure of At1g01230.1 and a spliced alignment produced by GenomeThreader of the Os07g26940.1 protein sequence against the final contig produced by an example SRAssembler run. At each of the portrayed rounds of recursion, the sequencing reads identified by SRAssembler as potentially part of a homologous locus are mapped (using Bowtie2 [33]) onto the final contig and visualized with the Integrative Genomics Viewer [34].

In the initial round, which uses the Os07g26940.1 protein sequence as the query to search the reads, SRAssembler finds reads that align to the exons. Because we simulated paired-end reads, both members of the pair are retained if either one of them aligns to the query. This leads to some low-depth coverage of the introns and untranslated regions (UTRs). During round 2, the contigs assembled from the reads found in round 1 are used to search for additional reads. By round 3 there is good read coverage of the full length of the target gene, and additional rounds find new reads at the contig borders that can extend its length. One observation from the results in Fig. 2 is that initial walking is very fast when connecting adjacent coding regions (i.e., exons), but extension of the contig boundaries is relatively slow. If a user’s region of interest is flanking, rather than part of, the coding sequence (e.g., promoter elements), more rounds of recursion are necessary.

### Comparison of SRAssembler to whole genome assembly

The ability to perform targeted local assembly is not useful if the results are low quality. To demonstrate the effectiveness of SRAssembler, we generated sets of simulated sequencing reads of Arabidopsis chromosome 1 with four depths of coverage ranging from 10X to 40X. Twenty different random “seeds” (numbers used to create reproducible pseudo-random output) were used with wgsim at each coverage level, producing a total of 80 sets of reads. We used SOAPdenovo2, the default assembler used by the SRAssembler workflow, to attempt full assemblies of Arabidopsis chromosome 1 from each of the 80 read sets.

The rice-homologs track from PlantGDB AtGDB [35] indicates that 20 loci within the first million bases of Arabidopsis chromosome 1 have rice homologs that are over 100 amino acids in length. These 20 rice protein sequences (Table 1) were used as queries for SRAssembler against the simulated read sets. The contigs produced by SRAssembler from each run were aligned using BLAST+ [36] against the full gene sequence of their respective Arabidopsis orthologs. The assembly contigs produced by SOAPdenovo2 were also searched using BLAST+ for the 20 aforementioned Arabidopsis genes.

**Table 1:** SRAssembly of 20 Arabidopsis genes

Rice query	Arabidopsis ortholog	Locus start	Locus end	Contig start	Contig end	Identity
Os03g02970.1	At1g01040.2	23121	31227	23070	32269	99.99
Os05g36260.1	At1g01050.1	31170	33171	30480	33907	99.97
Os04g02900.1	At1g01090.1	47234	49304	47020	49718	100
Os07g26940.1	At1g01230.1	97412	99240	96394	99684	99.85
Os08g06060.1	At1g01560.2	202103	204440	201313	205079	100
Os07g26660.1	At1g01620.1	225665	227543	224898	228253	100
Os02g44470.1	At1g01750.2	275188	276310	274408	277381	100
Os06g03660.2	At1g01820.1	296001	298334	295251	298848	100
Os09g34970.1	At1g01910.1	313101	316090	312601	316984	100
Os06g04000.1	At1g01940.1	322886	324917	321925	326047	99.98
Os06g04560.1	At1g01950.3	325316	330619	325316	331264	100
Os01g08450.1	At1g02130.1	399983	401919	399420	401973	100
Os12g18880.1	At1g02140.1	403100	404456	402439	405644	99.94
Os01g18860.1	At1g02500.1	518091	520495	517829	521327	100
Os03g19510.1	At1g02560.1	537740	540127	537158	540531	100
Os03g21940.1	At1g02780.1	607799	609534	607187	609669	100
Os03g22340.1	At1g02830.1	625084	625608	624076	626451	100
Os05g05260.1	At1g03190.1	775527	780062	774617	780567	99.87
Os04g52130.1	At1g03475.1	868853	871308	868050	872005	99.62
Os10g35370.1	At1g03630.1	907523	909650	907182	909855	99.93

Twenty Arabidopsis genes chosen from the first million base pairs of chromosome 1 were assembled using rice protein queries. “Locus start” and “Locus end” are the coordinates of the loci of the Arabidopsis genes, and “Contig Start” and “Contig End” indicate where the SRAssembler result aligns to Arabidopsis chromosome 1 with BLASTN

For both SRAssembler and SOAPdenovo2 the percent identity with the target reference sequence was over 98% in all cases, and over 99% when read coverage was 20X or higher. Assembly completeness of the target gene tended to correlate with coverage depth, but there was surprising variance between read sets generated from different random seeds, especially at 10X and 20X coverage, and increasing coverage depth did not universally improve assembly (Additional file 1: Figure S1). Our metric of comparison was the percentage of the target gene sequence that aligned to the assembled contigs produced by SRAssembler and SOAPdenovo2 for each read set. In 1062 out of 1600 cases (66.4%), SRAssembler and SOAPdenovo2 performed equivalently. In 271 cases (16.9%) SRAssembler produced a contig containing more of the target sequence, and in 267 cases (16.7%) SOAPdenovo2 was superior. SRAssembler was able to match or exceed the utility of a whole genome assembly in a total of 1333 cases (83.3%).

### Assembly of homologous loci from real data

In real-world experiments, NGS reads are typically not uniformly distributed over the genome sequence. The aforementioned 20 rice protein sequences were again used as queries for SRAssembler, this time to assemble sequencing reads from a Swedish accession of Arabidopsis [37].

We evaluated the performance of SRAssembler in this test by aligning the contigs it produced against the TAIR10 reference Arabidopsis genome using BLASTN. The results in Table 1 show that for each protein query, SRAssembler produced a contig that mapped to the chromosome region containing the expected Arabidopsis ortholog of the rice query. All 20 of these contigs share at least 99.5% identity with the Arabidopsis reference sequence and contain the entire homologous Arabidopsis locus.

As a test of application on a more complex genome, we also performed the reciprocal assemblies, using the proteins encoded by the Arabidopsis genes as probes for SRAssembler to build matching contigs from a set of reads from IRIS 313-11802, a cultivar of rice from the 3000 Rice Genomes Project [38]. Libraries ERR611677 to ERR611681 were used in this test, giving approximate 17X genome coverage. Contigs were built after several rounds of assembly and evaluated by the quality and extent of GenomeThreader spliced alignments of the corresponding known rice proteins. In 15 cases a contig covered over 90% of the rice protein (Table 2), clearly identifying a homologous gene, and in all cases at least part of a relevant gene was identified. Depending on the goals of the study, in practice a user can use any of the contigs as starting points for further assembly attempts, adding more reads data (if available) or extending contigs by further assembly rounds (SRAssembler will appropriately build on the previously generated results). A researcher may also consider designing primers for genomic PCR based on the assembly results in order to generate a more reliable sequence, targeted to the gene of interest.

**Table 2:** SRAssembly of rice homologs

Arabidopsis query	Rice homolog	Spliced alignment score	Homolog coverage
At1g01040.1	Os03g02970.1	0.997	1.000
At1g01050.1	Os05g36260.1	0.714	1.000
At1g01090.1	Os04g02900.1	0.997	1.000
At1g01230.1	Os07g26940.1	1.000	1.000
At1g01560.2	Os08g06060.1	0.967	1.000
At1g01620.1	Os07g26660.1	0.597	0.992
At1g01750.1	Os02g44470.1	0.780	1.000
At1g01820.1	Os06g03660.2	0.676	1.000
At1g01910.1	Os09g34970.1	0.889	0.843
At1g01940.1	Os06g04000.1	0.992	1.000
At1g01950.3	Os06g04560.1	0.492	0.800
At1g02130.1	Os01g08450.1	0.786	0.738
At1g02140.1	Os12g18880.1	0.778	0.981
At1g02500.1	Os01g18860.1	1.000	1.000
At1g02560.1	Os03g19510.1	0.820	0.908
At1g02780.1	Os03g21940.1	0.656	0.480
At1g02830.1	Os03g22340.1	0.696	0.841
At1g03190.1	Os05g05260.1	0.996	0.955
At1g03475.1	Os04g52130.1	1.000	0.913
At1g03630.1	Os10g35370.1	0.900	0.981

Twenty Arabidopsis proteins were used as queries into rice reads. The homologous rice proteins were spliced-aligned to the assembled contigs. The best result for each query is shown here

### Assembly of paralogous loci

SRAssembler often assembles multiple contigs, some of which are not ultimately of interest. These may contain distantly related genes, or just share a common domain with the query. Spliced alignment software such as GenomeThreader is used to identify the contigs labeled as “hits” against the query. In many cases, multiple hit contigs are the result of whole or partial genome duplication events. The ability to potentially identify and assemble paralogous (homologous due to duplication within a genome) as well as orthologous (homologous due to speciation) loci to genes of interest is an additional feature of the SRAssembler approach. This can be especially valuable in plants, which frequently undergo genetic duplication events.

The Arabidopsis Information Portal (Araport) [39] ThaleMine tool was used to identify genes paralogous to the 20 Arabidopsis loci from the previous section. Nineteen out of the 20 genes had at least one paralog identified in PANTHER version 11 [40]. Many of the contigs assembled by SRAssembler were identified by BLAST to correspond to one of these paralogs. Out of 295 Arabidopsis genes identified as homologous to the 20 rice queries, 79 (26.8%) genes were completely assembled. In many cases the assembled contigs covered only part of a paralogous locus. Beyond the 79 complete genes, 19 additional gene bodies (the region from the start codon to the stop codon, but not including UTRs) were fully assembled, and a total of 141 gene bodies had at least 50% of their length covered by a contig. Table 3 summarizes these results, and Additional file 2: Table S1 contains details for each paralogous gene individually.

**Table 3:** Summary of assembly of paralogous Arabidopsis genes

Arabidopsis target	Total paralogs in clade	Complete paralogs	Complete gene bodies	Gene bodies >50% assembled
At1g01040.2	6	1 (17%)	1 (17%)	1 (17%)
At1g01050.1	6	4 (67%)	4 (67%)	5 (83%)
At1g01090.1	3	1 (33%)	1 (33%)	1 (33%)
At1g01230.1	2	1 (50%)	2 (100%)	2 (100%)
At1g01560.2	29	3 (10%)	5 (17%)	17 (59%)
At1g01620.1	37	12 (32%)	14 (38%)	17 (46%)
At1g01750.2	12	9 (75%)	10 (83%)	10 (83%)
At1g01820.1	5	3 (60%)	3 (60%)	3 (60%)
At1g01910.1	3	1 (33%)	1 (33%)	3 (100%)
At1g01940.1	30	3 (10%)	6 (20%)	12 (40%)
At1g01950.3	64	1 (2%)	1 (2%)	10 (16%)
At1g02130.1	63	22 (35%)	30 (48%)	40 (63%)
At1g02140.1	1	1 (100%)	1 (100%)	1 (100%)
At1g02500.1	4	3 (75%)	4 (100%)	4 (100%)
At1g02560.1	10	2 (20%)	3 (30%)	3 (30%)
At1g02780.1	4	3 (75%)	3 (75%)	3 (75%)
At1g02830.1	3	3 (100%)	3 (100%)	3 (100%)
At1g03190.1	6	1 (17%)	1 (17%)	1 (17%)
At1g03475.1	2	2 (100%)	2 (100%)	2 (100%)
At1g03630.1	5	3 (60%)	3 (60%)	3 (60%)

Nineteen out of the 20 Arabidopsis gene “targets” have at least one annotated paralog. SRAssembler was able to completely assemble at least one additional paralog for 13 of those targets. In many cases in which the complete paralog was not assembled, contigs still covered a significant fraction of the gene body (region from the start codon to the stop codon). If further investigation of a clade is desired, the final contigs could be used as starting queries for new SRAssembler runs

### Intra-species comparison of gene homologs

Twenty representative cultivars from the 3000 Rice Genomes Project were selected to demonstrate the utility of SRAssembler for analyzing conservation of a gene within a species. The coding sequence of Os07g26940.1 was used as an example query. A homologous contig was successfully assembled from each of the cultivars. These contigs were aligned to the reference Os07g26940.1 gene sequence with MUSCLE [41] and show strong conservation in both exons and introns (alignment included as Additional file 4). Exon 1 has 99.5% identical sites, intron 1 has 98.9% identical sites, exon 2 has 100% identical sites, intron 2 has 96.8% identical sites, and exon 3 has 99.3% identical sites. The 5^′^-UTR has 92.1% identical sites, and the 3^′^-UTR has 99.7% identical sites. The 301 bp region of the multiple sequence alignment upstream of the Os07g26940.1 start site is also highly conserved, with 90.4% identical sites.

Further upstream of this point, nine of the cultivars diverge widely from the other eleven. Based on comparison to the rice reference Os-Nipponbare-Reference-IRGSP-1.0 [42], it appears that these cultivars likely share an insertion of at least 400 bp. This is notable because this variance from the reference is not reported in the Rice SNP-seek database [43] for any of the nine divergent cultivars (and obviously could not be reported, as the database data are derived from read mapping to the reference genome, which would necessarily miss anything longer than within-read length insertions or deletions).

### Assembly from short reads in RNAseq data

SRAssembler can also assemble contigs using sequencing reads from sources other than whole-genome sequencing experiments, such as RNAseq. RNAseq data from mouse skeletal muscle were used to assemble *Myf6*, a myogenic transcription factor gene [44]. Despite the reads being only 33 base pairs, SRAssembler was able to assemble a contig with 100% identity to the mRNA corresponding to the full length of the query protein.

### Running time

**Fig. 3:**
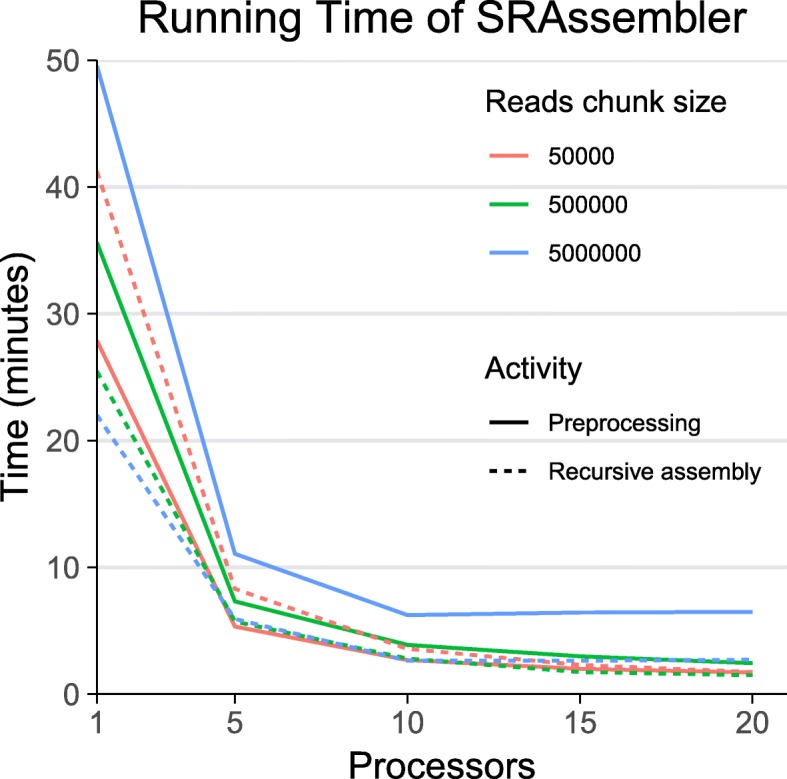
Running time of SRAssembler. SRAssembler was run for five rounds using the Os07g26940.1 protein sequence as a query for 42 million Arabidopsis genomic read pairs. The effect on running time of pre-existing preprocessed read chunks, the size of the read chunks, and the number of processors assigned to SRAssembler were each tested and are shown as the mean of three technical replicates. As the number of processors assigned to SRAssembler rises from one to 17, completion time drops dramatically. Using larger read chunks increases the time required for preprocessing but can decrease the time of chromosome walking up to some point. The speed gains from increasing numbers of processors show diminishing returns, and vanish if the number of processors exceeds the number of read chunks

Because SRAssembler directly assembles relatively short regions of interest, it takes far less time and computing power than a complete genome assembly. As a demonstration of the potential speed and resource use of SRAssembler, we used the rice protein Os07g26940.1 as a query for SRAssembler using 42 million Arabidopsis genomic read pairs from NCBI SRA ([20]) accession SRR519536. In these tests we ran the single-threaded SRAssembler with one processor and the openMPI-configured version of SRAssembler with 5, 10, 15, and 20 processors, and compared the amount of time spent on preprocessing reads and recursively assembling contigs (Fig. 3). Preprocessing in SRAssembler splits read files into conveniently manageable sizes, converts the read format from FASTQ to FASTA to minimize the storage footprint, and indexes the reads for speedy searching. If SRAssembler is rerun using the same sequencing data, the preprocessing step can be skipped, improving running time. This is useful when assembling several homologs from the same sequencing data, or when experimenting with different run parameters.

The number of chunks the read files are split into can impact the speed of an SRAssembler run. Using fewer, larger files makes read processing take longer, but can make subsequent runs faster, at least when using one processor. The advantages of parallelization disappear when the number of chunk files exceeds the number of processors available.

When SRAssembler was run on a single core with the default split file size of 500,000 reads, the execution time of this test averaged 61 min; this dropped to 4 min with 20 cores. A little more than half of this time was spent on the reads preprocessing stage. SRAssembler running time for a predetermined number of rounds is dependent on the number of processors and the size of the read libraries used, but is less predictable when stopping is based on completion of a contig that covers the query sequence.

## Discussion

SRAssembler is not the first software to use a recursive search approach to analyze NGS reads that have not yet been assembled. Tracembler used recursive BLAST searches within the NCBI Trace Archive, but was not capable of searching user-provided read libraries, and used a less sophisticated approach to selecting and assembling reads [22]. The Mapsembler program is a tool targeting specific biological events such as SNPs, splicing events, or gene fusion [45]. Although Mapsembler also uses an iterative search algorithm as in Tracembler and SRAssembler, it is not designed to assemble homologous loci, does not use paired-end reads, and will not accept a protein query sequence. A tool called MITObim [46] uses a “baiting and iterative mapping” strategy similar to our “in silico chromosome walking” to assemble mitochondrial genomes, but it is not designed to assemble regions homologous to generic protein or DNA sequences.

## Conclusions

SRAssembler offers a fast, efficient way to assemble whole-genome sequencing reads into contigs containing regions of interest, and we expect this approach to be useful to biology researchers for a variety of purposes. One obvious use case is a researcher who is interested in the sequence of a specific gene in an organism that does not have an assembled genome. This could be a particularly interesting individual or population from a species with an existing reference genome, or perhaps a member of a previously unsequenced species. Rather than spending time and computational resources on whole-genome assembly (a process which still intimidates many biologists), SRAssembler is intended to allow the researcher to assemble the sequence they care about and move forward with their research questions. Our testing suggests that the assembly of a target homologous gene has accuracy similar to full genome assemblies by modern software, while being much faster and requiring fewer resources.

The speed and computational resource advantages of SRAssembler over whole-genome assembly become even more apparent when trying to scale up an experiment. If a researcher is interested in a specific gene family, not within a single individual, but in each of 500 members of a population, computational resources and time are likely to be more prohibitive than the falling costs of NGS technology. This is especially true if the researcher is using pre-existing sequencing reads.

Whether or not an SRAssembler assembly attempt succeeds depends on the specifics of the application, including factors such as available read depth, intron content and length of the target gene, repetitive sequence content of the target region, and degree of similarity between the probe and target sequences. In favorable conditions, a quick success is likely. In difficult conditions, a variety of SRAssembler options allow flexible use of the program that can often still lead to success.

Collections like the human 1000 Genomes project [47] and the 3000 Rice Genomes Project mean that researchers can perform new bioinformatic experiments without needing to collect new data. Sequencing reads collections have already been used to create tools like the Rice SNP-seek database, which mapped sequencing reads onto the rice Nipponbare reference genome to detect SNPs and other small variants in each of the sequenced cultivars [42]. However, as demonstrated in one of our experiments, this method is not sufficient to detect larger variants such as long indels or chromosome rearrangements. The SRAssembler strategy of recursively searching for reads that match the region of interest is not biased by an existing reference sequence, and can lead to discoveries that reference-mapping alone cannot.

Ongoing and future massive sequencing projects (ultimately, the Earth BioGenome Project [21]) will generate unprecedented opportunities for detailed comparative genomics studies. SRAssembler should be a useful tool to aid in the transformation of such sequence data into knowledge.

## Availability and requirements

**Project name:** SRAssembler

**Project home page:** https://github.com/BrendelGroup/SRAssembler

**Operating system:** UNIX-like

**Programming language:** C++

**Other requirements:** Singularity v2.4.2+, OR Vmatch v2.3.0, SOAPdenovo2 v2.04, ABySS v2.1.0, GeneSeqer, GenomeThreader v1.7.0+, and SNAP v2006-07-28

**License:** GNU GPL-3.0

**Any restrictions to use by non-academics:** None

## Additional files

Additional file 1 Figure comparing assembly of target genes from simulated reads as a factor of read coverage depth. Twenty Arabidopsis genes were assembled from 20 different sets of simulated reads (“seeds”), at four different read coverage depths. Although the majority of gene/seed combinations showed improved assembly of the gene with increasing coverage depth, each gene had at least one seed that produced a worse result at higher coverage depth than it had at a lower depth (demonstrated by negative line slopes). (PDF 13 kb)

Additional file 2 Table of Arabidopsis orthologs to rice queries and their aligned contigs. The contigs from twenty SRAssembler runs using rice proteins against Arabidopsis genomic sequencing reads were aligned with blastn against the Arabidopsis reference genome. The Arabidopsis orthologs (“Atgene”), their locations(“GeneStart” and “GeneStop”), and the beginning and end of their coding sequences (“ORFstart” and “ORFstop”) were identified with Araport Thalemine. If a contig from the SRAssembler run using the homologous rice protein (“Osprobe”) overlapped with an Arabidopsis gene location, this is noted in the “Contig” column, as is the percent identity with (“Identity”) and location on (“ContigStart” and “ContigStop”) the reference genome. The length of overlap between the contig and each gene locus (“ContigGeneOverlap”) and coding region (“ContigORFOverlap”) are noted, as are the fraction of the total gene and coding region lengths (“GeneCoverage” and “ORFCoverage”). (TSV 30 kb)

Additional file 3 Scripts and files for complete replication of results. Unpacking this tarball will create directories and scripts that, when executed, will download all necessary reads and perform all of the experiments described in this manuscript. The scripts will reproduce all of the tables and figures in this manuscript except for the workflow diagram. (TAR 340 kb)

Additional file 4 FASTA-format alignment of hit contigs from 20 rice cultivars. The Os07g26940.1 CDS sequence was used as the SRAssembler query against sequencing reads from 20 rice cultivars. The hit contigs were aligned with the reference genomic sequence for Os07g26940.1 using MUSCLE. (MUSCLE 154 kb)

## Data Availability

The sequence for the TAIR10 *Arabidopsis thaliana* chromosome 1 reference sequence is available from The Arabidopsis Information Resource [48]. The *Arabidopsis thaliana* sequencing reads dataset analyzed during the current study are available in the European Bioinformatics Institute SRA database, accession SRR519536 [37]. The cultivars of rice used in the current study were: IRIS 313-11737, IRIS 313-10603, IRIS 313-10177, CX357, IRIS 313-11643, IRIS 313-11671, IRIS 313-11723, IRIS 313-11736, IRIS 313-11790, IRIS 313-11794, IRIS 313-11800, IRIS 313-11812, IRIS 313-11924, IRIS 313-15910, IRIS 313-8326, IRIS 313-8493, IRIS 313-8658, IRIS 313-8665, IRIS 313-8669, and IRIS 313-11802. Information on where to find their sequencing reads is available through the 3000 Rice Genomes Project [38]. The mouse sequencing reads dataset analyzed during the current study are available in the European Bioinformatics Institute SRA database, accessions SRR001361 and SRR001362 [44]. All other data generated or analyzed during this study are included in this published article and its supplementary information files.

## Abbreviations

MPI: Message passing interface
NGS: Next-generation sequencing
SRA: Sequence read archive
UTR: Untranslated region
