# Supplementary figures and images for: SRAssembler: Selective Recursive local Assembly of homologous genomic regions
Source: BMC Bioinformatics. 2019 Jul 2;20:371. doi: 10.1186/s12859-019-2949-4 (PMC6604332; doi:10.1186/s12859-019-2949-4)

# Assembly of target gene as a factor of read coverage depth

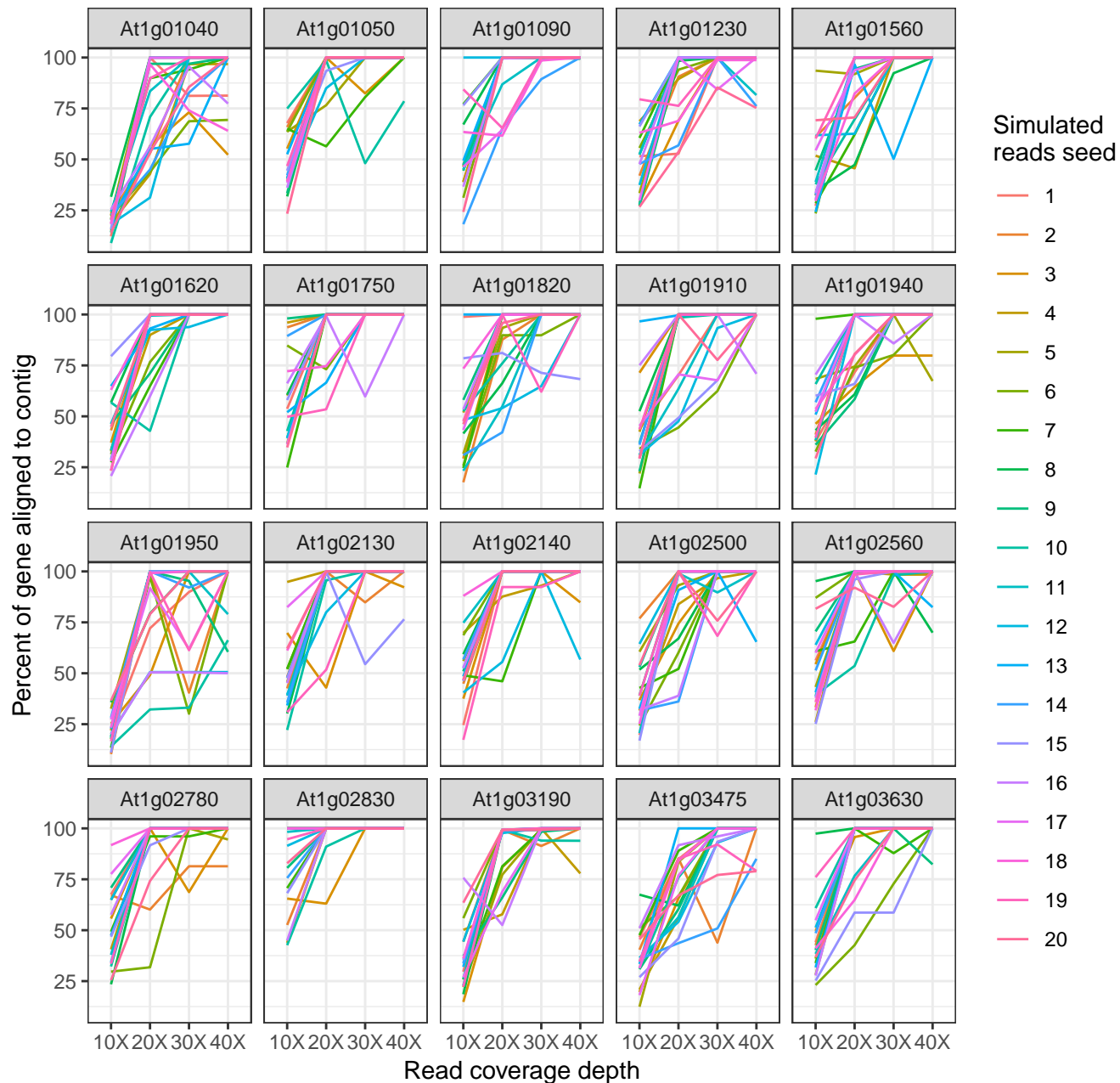

Supplement: Supplementary file 1 — Figure comparing assembly of target genes from simulated reads as a factor of read coverage depth. Twenty Arabidopsis genes were assembled from 20 different sets of simulated reads (“seeds”), at four different read coverage depths. Although the majority of gene/seed combinations showed improved assembly of the gene with increasing coverage depth, each gene had at least one seed that produced a worse result at higher coverage depth than it had at a lower depth (demonstrated by negative line slopes). (PDF 13 kb) [file 12859_2019_2949_MOESM1_ESM.pdf]
